# Effect of Salinity on Silica Nanoparticle Adsorption Kinetics and Mechanisms for Fluid/Rock Interaction with Calcite

**DOI:** 10.3390/nano9020213

**Published:** 2019-02-06

**Authors:** Aly A. Hamouda, Rockey Abhishek

**Affiliations:** Institute of Energy and Petroleum Technology, University of Stavanger, 4036 Stavanger, Norway; rockey.abhishek@uis.no

**Keywords:** chalk, silica NP, calcite dissolution, adsorption kinetics, intraparticle diffusion, kinetics of NP/chalk interaction, interaction forces in presence of salt

## Abstract

This study addresses the kinetics of silica nanoparticle adsorption on calcite from a solution at three salinities: deionized water (DIW), synthetic seawater (SSW), and low salinity water (LSW). The nanoparticle adsorption mechanisms and the effects on calcite dissolution are addressed. It was shown that nanoparticle adsorption was best described with the second-order-kinetic model and that silica nanoparticle adsorption reduced calcite dissolution. This was confirmed by measuring the Ca^2+^ ion concentration, the pH, and by estimating the amount of calcite dissolved. This is an important conclusion of this work, especially as LSW as an enhanced oil recovery technique is a candidate for use in chalk fields. Less formation damage/dissolution of chalk when silica nanoparticles are combined with LSW can lower the risk of reservoir subsidence. Intraparticle diffusion and the pseudo-second-order models, indicated a reduction in the adsorption rate with increasing nanoparticle concentration in LSW. This is explained by possible repulsive forces among the nanoparticles as they diffuse from the bulk fluid onto the calcite surface. Ion charges reduce the repulsion among the nanoparticles through shielding. However, an increasing nanoparticle concentration reduces the shielding efficiency by the ions. Estimates of the surface forces confirmed that nanoparticle–mineral interaction is less attractive in LSW as compared to SSW and DIW.

## 1. Introduction

Nanofluids are colloidal dispersions of nanoparticles with sizes below 100 nm dispersed in a suitable medium. Over the past decade, nanofluids have attracted a lot of attention for enhanced oil recovery (EOR) from petroleum reservoirs [1,2,3,4]. The effectiveness of nanoparticles (NP) for enhancing oil recovery has been investigated by many researchers [2,5,6,7,8,9,10,11]. Among the various metal oxide nanoparticles, silica has emerged as a promising material for EOR due to: (1) ease of surface functionalization, (2) good transport properties in the reservoir, and (3) wettability change due to adsorption of silica nanoparticles on the rock surface [12,13,14]. In addition, silica nanoparticles have found applications in fields such as CO_2_ reforming [15], removal of organic and inorganic pollutants [16], drug delivery [17], environmental materials [18], among others. Since the incremental oil recovery obtained from the application of silica nanoparticles is generally attributed to the wettability alteration, the adsorption of nanoparticles on the rock surface is of primary importance for modifying the rock surface from an oil-wet to a water-wet state.

While some studies have addressed the adsorption behavior of nanoparticles in sandstone reservoirs [12,19,20,21,22,23], few investigations have addressed the applicability of nanoparticles to carbonate reservoirs [24,25,26,27,28]. Nazari Moghaddam et al. [29] addressed the applicability of nanoparticles in altering the wettability of carbonate reservoirs. Al-Anssari, et al. [30] reported that silica nanoparticle adsorption on the calcite surface is irreversible and it can cause wettability alteration from an oil/mixed-wet to water-wet state. Their research group also reported that the silica nanofluid treatment was more effective at elevated temperatures [24]. Monfared, et al. [31] studied silica nanoparticle adsorption on calcite surfaces and the effect of salinity and pH on the adsorption process. However, the adsorption of silica nanoparticles on the calcite mineral, which is the major constituent of chalk reservoirs, is not well understood.

Chalk reservoirs are highly porous but have low permeability. Chalk reservoirs have pore throats in the order of 0.2 μm [32]. The use of micro particles of silica could lead to a blockage of the pore throats and hence nanoparticles with particle size of less than 100 nm are ideal for chalk reservoirs. Previous work in our lab [33] addressed the adsorption of silica nanoparticles dispersed in different brines on chalk surfaces and their effect on fluid/rock interaction especially during combined nanoparticle and low salinity water injection. Low salinity water flooding has emerged as a cheap and environmentally friendly technique for improving oil recovery [34,35,36,37,38,39,40,41]. Increased calcite dissolution induced by the interaction between the injected low salinity water and calcite [42,43,44,45] during flooding may lead to a loss of rock integrity [46]. However, we found that silica nanoparticles could reduce calcite dissolution by ≈30% induced by low salinity flooding of chalk, in addition to increasing oil recovery that can be achieved by low salinity flooding alone [33]. The adsorption behavior of nanoparticles was studied during the flooding process. The present work focuses on the kinetic aspects of the adsorption process on the calcite mineral. Batch adsorption experiments were carried out at three salinities: deionized water (no added salts), seawater (high salinity) and low salinity water (at 1:10 seawater dilution). Additionally, the calcium ion concentration and pH were tracked during the batch adsorption experiments to address the effect of nanoparticle adsorption on calcite dissolution.

## 2. Materials and Methods

The silica nanoparticles (DP9711) were obtained at 30% weight (wt.) concentration from Nyacol Nano Technologies dispersed in deionized water (DIW). The nanofluids were prepared by diluting the stock dispersion with appropriate fluids. While aggregation is an issue with nanoparticles in general, the stability of the used nanoparticles (DP9711) in DIW, synthetic seawater (SSW), and low salinity water (LSW) with 1:10 SSW dilution has been investigated previously [33]. We found that after three months, particle size measurements were close to the initial measured values (within 5 nm). In addition, the nanofluids remained visually clear with no sign of sedimentation. Calcite mineral powder of analytical grade was acquired from Riedel-de Haen AG (Hannover, Germany). The specific surface area of the calcite powder (0.23 m^2^/g) has been determined previously in our lab [34]. SSW (pH 7.45) and LSW (pH 7.32) were the brines used in this study. LSW at 1:10 dilution was used based on our previous work for the assessment of the best dilution performance as an EOR method [40,46]. The ionic composition of the brines is listed in Table 1.

The nanofluids were prepared at 1 g/L nanoparticle concentration in DIW, LSW, and SSW. The average particle size (hydrodynamic radius) and zeta potential (Smoluchowski model) of the silica nanoparticles were measured previously [33]. The zeta potential (Smoluchowski model) of calcite mineral powder dispersed in different fluids was also measured using a Zetasizer Nano ZSP from Malvern Instruments (Malvern, UK). The values are listed in Table 2.

### 2.1. Adsorption Experiments

Five grams of calcite powder was dispersed in 30 mL of nanofluid. The nanofluid was prepared at a predetermined nanoparticle concentration and dispersion fluid salinity. The nanofluid-calcite dispersion was placed in a 50 mL capped centrifuge tube. The tube containing the nanofluid and the mineral was then agitated on a rotary agitator for the desired length of time. At the end of the period, the mineral was removed from the fluid by centrifuging at 10,000 rpm and decanting the supernatant fluid. The supernatant fluid was then filtered through a 0.22 μm filter, which allowed the nanoparticles to pass through but not the larger calcite mineral particles. The remaining concentration of the nanoparticles in the supernatant was determined by their absorbance in a dual beam UV/Vis (Ultraviolet–visible) spectrophotometer (UV/Vis 1800 spectrophotometer from Shimadzu Corporation, Kyoto, Japan) at 240 nm wavelength against DIW, comparing it with the calibration curves and making baseline corrections. The supernatant nanoparticle concentration was then used to estimate the amount of nanoparticles (adsorbate) adsorbed on the known amount of calcite mineral (adsorbent). A series of adsorption experiments were performed with increasing time until equilibrium adsorption was reached. To address the extent of calcite mineral dissolution, the pH of the supernatant was measured using S220 SevenCompact^TM^ pH/ion meter by Mettler-Toledo International Inc (Columbus, OH, USA) calibrated with buffers of pH 7 and 10.1. The concentration of the Ca^2+^ in the supernatant fluid was determined by Ion Chromatography (IC) using a Dionex ICS-5000 ion chromatograph from Thermo Fisher Scientific (Waltham, MA, USA). Additionally, mineral dispersions prepared in different fluids (without nanoparticles) were also analyzed for Ca^2+^ concentration and pH to obtain a baseline for comparison.

### 2.2. Surface Forces

The interaction energies between the nanoparticle and calcite minerals affect the adsorption of nanoparticle on the mineral. The theory of surface forces can be utilized to calculate the interaction energies between the nanoparticles and calcite minerals based on the Derjaguin–Landau–Verwey–Overbeek (DLVO) theory. As a result of the size difference between the nanoparticles and mineral, the curvature of the mineral surface may be neglected and the interactions can be modeled based on Sphere–Plate collector geometry. The net interaction (*V_t_*) as a function of separation distance (*h*) is the sum of London–van der Waal interaction (*V_LVA_*) and Electric double layer interaction (*V_EDLR_*), which can be calculated as:

(1) $$V_{t}\left(h\right)=V_{LVA}\left(h\right)+V_{EDLR}\left(h\right),$$

where *k_B_* is the Boltzmann constant (1.38 × 10^−23^ J K^−1^) and *T* is temperature. The contributions, as a result of the different interactions in Equation (1) based on the constant potential approach, can be calculated as follows [31,47,48]:

(2) $$V_{LVA}\left(h\right)=-\frac{A_{132}}{6}\left[\frac{a_{p}}{h}+\frac{a_{p}}{h+2a_{p}}+ln\left(\frac{h}{h+2a_{p}}\right)\right],$$

(3) $$\begin{array}{c} V_{EDLR}\left(h\right)=\pi \varepsilon_{0}\varepsilon_{3}\kappa \left(\zeta_{p}^{2}+\zeta_{s}^{2}\right)\int_{0}^{a_{p}}(-coth[\kappa \left(h+a_{p}-a_{p}\sqrt{1-{\left(h/a_{p}\right)}^{2}}\right)]+\\ coth\left[\kappa \left(h+a_{p}+a_{p}\sqrt{1-{\left(h/a_{p}\right)}^{2}}\right)\right]+\frac{\zeta_{p}\zeta_{s}}{\zeta_{p}^{2}+\zeta_{s}^{2}}csch\left[\kappa \left(h+a_{p}-a_{p}\sqrt{1-{\left(h/a_{p}\right)}^{2}}\right)\right]-\\ \frac{\zeta_{p}\zeta_{s}}{\zeta_{p}^{2}+\zeta_{s}^{2}}csch\left[\kappa \left(h+a_{p}+a_{p}\sqrt{1-{\left(h/a_{p}\right)}^{2}}\right)\right])r.dr, \end{array}$$

where *a_p_* is the silica particle radius (m) and *A*_132_ is the Hamaker’s constant calculated according to the Lifshitz theory based on the refractive indices, dielectric constants, and the temperature [48]:

(4) $$A_{132}\approx \frac{3}{4}K_{b}T\left(\frac{\varepsilon_{1}-\varepsilon_{3}}{\varepsilon_{1}+\varepsilon_{3}}\right)\left(\frac{\varepsilon_{2}-\varepsilon_{3}}{\varepsilon_{2}+\varepsilon_{3}}\right)+\frac{3h_{o}v_{e}}{8\sqrt{2}}\left(\frac{\left(\eta_{1}^{2}-\eta_{3}^{2}\right)\left(\eta_{2}^{2}-\eta_{3}^{2}\right)}{{\left(\left(\eta_{1}^{2}+\eta_{3}^{2}\right)\left(\eta_{2}^{2}+\eta_{3}^{2}\right)\right)}^{\frac{1}{2}}({\left(\eta_{1}^{2}+\eta_{3}^{2}\right)}^{\frac{1}{2}}+\left(\eta_{2}^{2}+\eta_{3}^{2}{)}^{\frac{1}{2}}\right)}\right),$$

where *ε*_1_(8), *ε*_2_(4.5), and *ε*_3_(80) represent the static dielectric constants of the interacting species (mineral and nanoparticle) and the intervening media (water), respectively [49]. *η*_1_(1.66) [50], *η*_2_(1.45) [51], and *η*_3_(1.33) [52] represent the refractive indices at 0.5876 μm wavelength of the interacting species (mineral and nanoparticle) and the intervening media (water), respectively. The refractive index can vary by approximately 7.9 × 10^−3^ between fresh water and salt water and its effect has been neglected [53]. Hence, in this study, the same value of refractive index is assumed for all mediums. *h_o_* is the Planck’s constant (6.626 × 10^−34^ J s) and *v_e_* is the main electron absorption frequency in the ultraviolet region and its value is between 3–5 × 10^15^ s^−1^ [50]. The permittivity of free space *ε*_0_: 8.854 × 10^−12^ C^2^ J^−1^ m^−1^*. ζ_p_* and *ζ_s_* are the surface potentials of the nanoparticles and minerals, respectively, which can be considered as the zeta potential. The estimation of the surface forces in this study was performed at 25 °C. For DIW, the inverse Debye length can be taken as (9.6 × 10^−7^)^−1^ m^−1^ [54]. For the saline mediums, the inverse Debye length (*κ^−^*^1^) depends on the salinity of the intervening medium (LSW/SSW) and can be calculated as:

(5) $$\kappa^{-1}=\sqrt{\frac{\varepsilon_{0}\varepsilon_{w}k_{B}T}{2e^{2}I}},$$

where *e* is the elementary charge of an electron (C), *k_B_* is the Boltzmann constant, and *I* is the ionic strength of the medium:

(6) $$I=\frac{1}{2}\sum c_{i}Z_{i}^{2},$$

where *c_i_* is the ion concentration of the *i*th species and *Z_i_* is the valence number of the *i*th species as listed in Table 1. The data used for the surface force calculation has been has been listed in Table 2. Finally, the total non-dimensionalized interaction energy (*V_t,ND_*) can be calculated as follows:

(7) $$V_{t,ND}\left(h\right)=\frac{(V_{LVA}\left(h)+V_{EDLR}\left(h\right)\right)}{k_{B}\times T}.$$

## 3. Results and Discussions

Low salinity water injection has emerged as an EOR technique for chalk reservoirs [36,37,38,39]. LSW has also been shown to promote calcite dissolution [40,46] which can affect chalk matrix integrity and lead to subsidence. However, our previous work [33] showed that silica nanoparticles have a tendency to adsorb on calcite surface and reduce the solubility of calcite by about 30% during combined silica nanoparticles and low salinity flooding of chalk. This work addresses nanoparticle adsorption kinetics on calcite and its effect on fluid/mineral interaction. The adsorption of nanoparticles dispersed in water at three salinities (DIW, LSW, and SSW) and its influence on calcite dissolution mechanisms were investigated. The nanoparticle concentrations used were 1 g/L for all the fluids except an additional concentration of 1.5 g/L that was used in the case of LSW. The LSW used in this work was SSW diluted 1:10 by DIW. The selection of the LSW composition was based on our previous work as well as that of other researchers based on its performance as an EOR injection fluid.

### 3.1. Adsorption Kinetics

**Figure 1:**
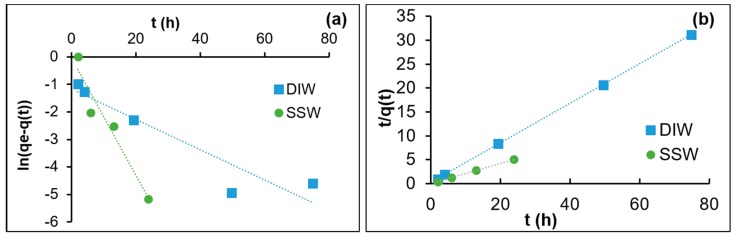
Data fit with kinetic models for the adsorption of nanoparticles on calcite in DIW and SSW: (**a**) pseudo-first-order (**b**) pseudo-second-order models.

The nanoparticle adsorption data obtained from the experiments described in Section 2.1 were fitted to pseudo-first-order and pseudo-second-order models to address the order of the adoption process. The linearized form of the pseudo-first-order and second-order models can respectively be expressed as [31,55]:

(8) $$\ln\left(q_{eq}-q\left(t\right)\right)=ln(q_{eq})-k_{1}t,$$

(9) $$\frac{1}{q\left(t\right)}=\frac{1}{k_{2}q_{eq}^{2}}+\frac{t}{q_{eq}},$$

where *q(t)* and *q_eq_* are the experimentally obtained data of nanoparticle adsorption (mg/g) on calcite at a given time (*t*) and equilibrium, respectively. *k*_1_ (1/h) and *k*_2_ (g/mg h) are the respective rate constants. The linear fits for kinetic adsorption data in DIW and SSW are shown in Figure 1. Figure 1a,b, examine the linearity fit with the data by *ln*(*q_eq_-q*(*t*)) vs. *t* and *t/q(t)* vs. *t*, respectively, for pseudo-first and pseudo-second-order models. The slope and the intercept were used to estimate the rate constants and equilibrium adsorption for both models (Table 3). It is shown in Figure 1a and Table 3 that the *R*^2^ correlation values of the linear fits are poor (0.88–0.94) for both DIW and SSW. Additionally, the model estimated equilibrium adsorption varies significantly from the experimentally observed level of equilibrium adsorption. Therefore, it may be concluded that the pseudo-first-order model does not describe the adsorption process well. However, the fits for adsorption in both DIW and SSW are excellent for the pseudo-second-order model (Figure 1b). The *R*^2^ values are close to 1 and the model estimated equilibrium adsorption agrees well with the experimental data (Table 3). This indicates that the pseudo-second-order model best describes the adsorption of silica nanoparticle on the calcite surface. It is interesting to see that at elevated salinity, SSW, the adsorption rate is ≈3 times higher than that for DIW and the equilibrium adsorption almost doubled.

**Figure 2:**
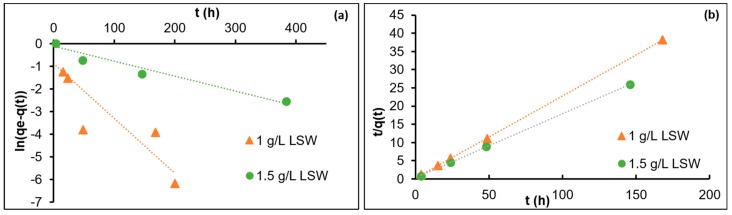
Data fit with kinetic models for the adsorption of nanoparticle on calcite in LSW: (**a**) pseudo-first-order (**b**) pseudo-second-order models.

To address the adsorption of nanoparticles in LSW, two sets of kinetic adsorption experiments were performed with two nanoparticle concentrations, 1 and 1.5 g/L, while the amount of the calcite was kept constant. It was shown that the adsorption data with the pseudo-second-order model for both nanoparticle concentrations fitted well. Figure 2a,b and Table 3 show the data fit, fitting coefficients, and the estimated equilibrium adsorption. It is shown in Figure 2a and Table 3 that *R*^2^ for the first order are poor (0.9–0.93) for both concentration of nanoparticles in LSW and the model estimated equilibrium adsorption varies significantly from the experimentally observed level of equilibrium adsorption. It is therefore concluded that similar to the adsorption of nanoparticles from DIW and SSW, pseudo-second-order models describe the adsorption process well, as *R*^2^ ≈ 1 for both the concentrations and the model estimated equilibrium adsorption is close to the experimental equilibrium adsorption. It is interesting to note that as the nanoparticle concentration increases from 1 to 1.5 g/L, the rate of adsorption decreases from 0.191 to 0.11 g/mg hr. In addition, the adsorption rates in LSW (for both concentrations) are lower than the rate estimated for DIW and SSW. This observation is discussed in the following section. From the kinetic adsorption data discussed so far, it may be concluded that the adsorption for silica nanoparticles from the three dispersing fluids (DIW, SSW, and LSW) onto the calcite surface is a second-order process. The adsorption mechanism is discussed in the following section.

### 3.2. Intraparticle Diffusion Model (IPD)

The proposed model of Weber and Morris [56] has been applied in previous studies to understand adsorption mechanisms. The linear relationship between *q(t)* and *t*^0.5^ indicates the contribution of intraparticle diffusion. Wu et al. [57] used the fractional approach to equilibrium change to determine the IPD contribution to the adsorption kinetics as follows:

(10) $$q_{t}=K\;t^{0.5}+C,$$

(11) $$q_{eq}=K{\;t}_{eq}^{1.5}+C.$$

Rearrangement yields,

(12) $$\frac{q_{t}}{q_{eq}}=1-R_{i}\left[1-{\left(\frac{t}{t_{eq}}\right)}^{0.5}\right],$$

where

(13) $$R_{i}=K\frac{t_{eq}{}^{0.5}}{q_{eq}},$$

Here, *R_i_* is defined as the initial adsorption factor, *K* (mg/g h^0.5^), *q_t_* (mg/g), *q_eq_* (mg/g), t (hr), *t_eq_* (hr), and *C* (mg/g) are the intraparticle diffusion rate, adsorbed amount at time t, adsorbed amount at equilibrium, time (h), the time to reach equilibrium, and initial adsorption amount (intercept). *R_i_* may also be expressed as the ratio of the initial adsorption to equilibrium adsorption amounts, which is used in this work

(14) $$R_{i}=1-\frac{C}{q_{eq}}.$$

From Equation (14), if *C* = 0, that means there is no initial adsorption in the system.

**Figure 3:**
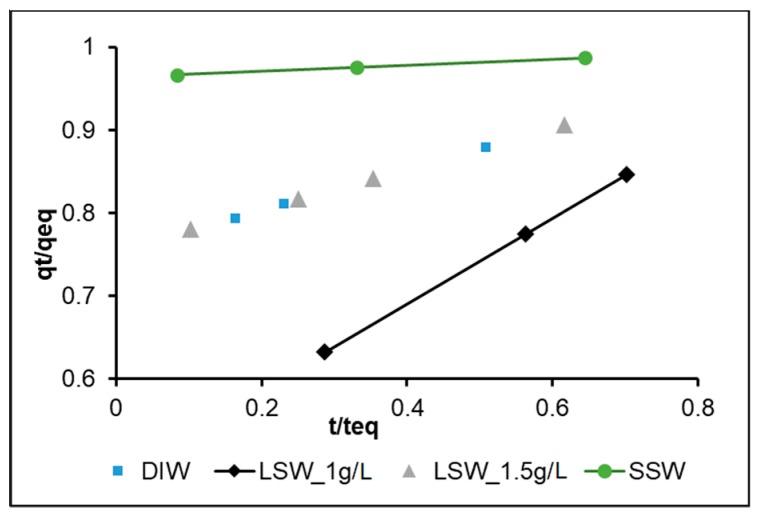
Non-dimensional intraparticle diffusion model for adsorption characteristic curves of the four tested systems with the dispersed silica nanoparticles.

Figure 3 shows the characteristic curves for DIW (nanoparticle conc 1 g/L), LSW (nanoparticle conc 1 g/L), LSW (nanoparticle conc 1.5 g/L), and SSW (nanoparticle conc 1 g/L) systems. Table 4 shows the classified adsorption characteristic according to Wu et al. [57]. In the case of DIW, LSW (1 g/L) and LSW (1.5 g/L) adsorption is classified as strong initial adsorption. That is, all the systems follow strong initial adsorption behavior except SSW (1 g/L), which is shown to be approaching complete initial adsorption, where q_e_ is almost equal to C (initial adsorption amount). In addition, for SSW, the time to reach equilibrium is almost 50% less than that for the other systems.

The reduced *R_i_* in LSW, as the nanoparticle concentration increases from 1 to 1.5 g/L to almost half may be explained by repulsive forces among the nanoparticles as they diffuse from the bulk fluid towards the calcite surface. In other words, the effect of ion charges could help to reduce the repulsive forces. However, the efficiency of the ion charges in shielding nanoparticles and reducing the repulsive forces among them is reduced as the nanoparticle concentration increases. This may also explain the lower adsorption rate observed for LSW with nanoparticles at 1.5 g/L during our investigation of the adsorption kinetic order in the earlier section.

Another interesting observation is that *R_i_* is almost equal in both DIW and LSW (1.5 g/L), which may support the above hypothesis. That is to say, in the presence of dissolved salts, the ions work as a barrier reducing the adsorption rate and in the absence of salt ions (DIW) the repulsive force among the nanoparticles reduces the adsorption rate. This is an interesting phenomenon worth further investigation.

**Figure 4:**
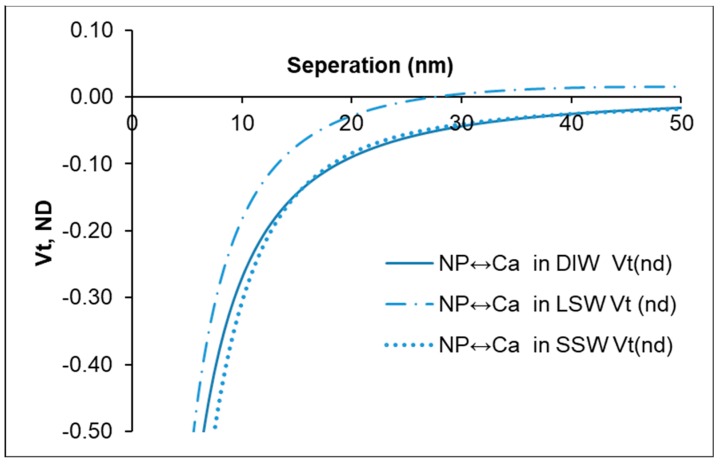
Total interaction energy between nanoparticles (1 g/L) and calcite (Ca) mineral in DIW, SSW, and LSW.

It is shown in Figure 4 that the total interaction energies, estimated by the DLVO theory, between nanoparticle and calcite mineral remain attractive at all separations in DIW and SSW. However, in the case of LSW, the interaction energy is shown to be less attractive and becomes slightly repulsive at around 30 nm separation. In other words, the LSW system involves more repulsive conditions compared to the SSW and DIW systems.

### 3.3. Fluid/Mineral Interaction

Two main chemical processes may take place between fluids and mineral (CaCO_3_). Those are dissolution and adsorption, as presented below:

(15) $${\mathrm{CaCO}}_{3}+H_{2}O⇌{\mathrm{Ca}}^{2+}+{\mathrm{HCO}}_{3}^{-}+{\mathrm{OH}}^{-},$$

(16) $$2{\mathrm{CaCO}}_{3}+H_{2}O+\mathrm{NP}⇌{\mathrm{CaCO}}_{3}-\mathrm{NP}+{\mathrm{Ca}}^{2+}+{\mathrm{HCO}}_{3}^{-}+{\mathrm{OH}}^{-}.$$

As shown in Equation (15), dissolution of calcite increases the pH. The adsorption process may be presented by Equation (16), where OH^−^ and HCO_3_^−^ are among the reaction products. The above two reactions indicate an increase in the fluids’ pH due to calcite dissolution.

**Figure 5:**
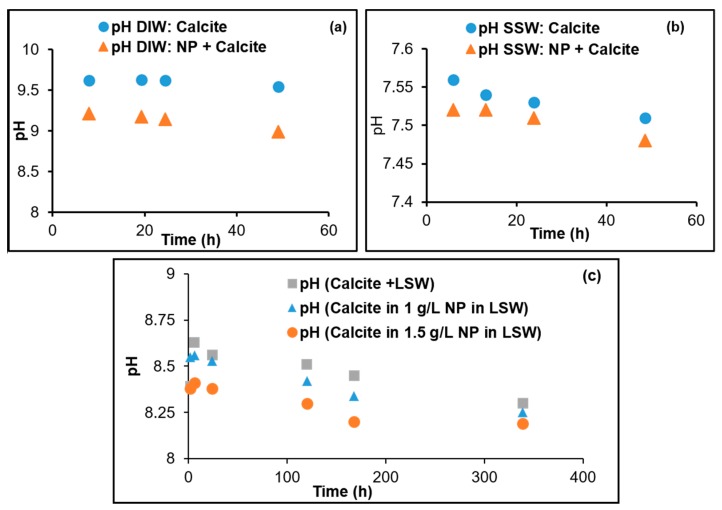
pHs of (**a**) DIW, (**b**) SSW, and (**c**) LSW (1 and 1.5 g/L) as a function of time during the kinetic adsorption tests.

The pH values with the dispersed nanoparticles in DIW, LSW, and SSW are 6.0, 7.2, and 7.3, respectively. The pH ranges vary depending on the fluid in which the adsorption and dissolution are taking place. That is, the pH is not controlled but the pH was monitored during the progression of the experiments. The changes in the pH with time during the experiments for the different dispersing fluids with and without nanoparticles are shown in Figure 5. The order of the pH values from highest to lowest for nanoparticle dispersing fluids are DIW > LSW(nanoparticle conc 1 g/L) > LSW(nanoparticle conc 1.5 g/L) > SSW. Generally, in all cases, during the dissolution/adsorption processes the pH declines. However, the changes are within about 0.3 pH units. The reduction may be explained by the formation of silanol, as a result of the dissociation of water molecules to form silanol groups and reduce the pH [58]:

(17) $$-\mathrm{SiOH}⇌-{\mathrm{SiO}}^{-}+H^{+}.$$

In spite of the reduction of the pH, the dissolution of calcite is also reduced (this is discussed later), contrary to what is expected. There are two factors which contribute to less dissolution. The first is that the pH balance between calcite dissolution and formation of silanol shows an insignificant decrease in the pH, as discussed above. The second factor is the adsorption of the nanoparticles on the calcite surface which affects the dissolution and perhaps the formation of silanol.

**Figure 6:**
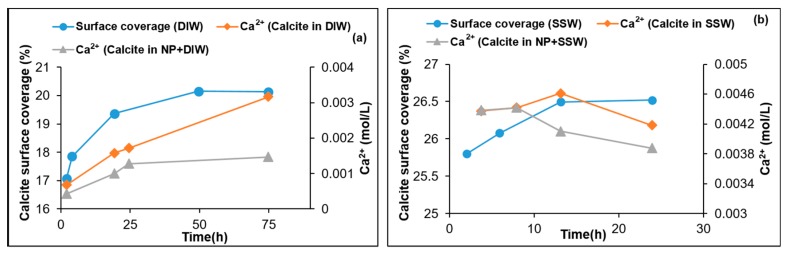
Supernatant Ca^2+^ concentrations with and without nanoparticles and the estimated surface coverage by nanoparticles (**a**) DIW and (**b**) SSW fluids.

**Figure 7:**
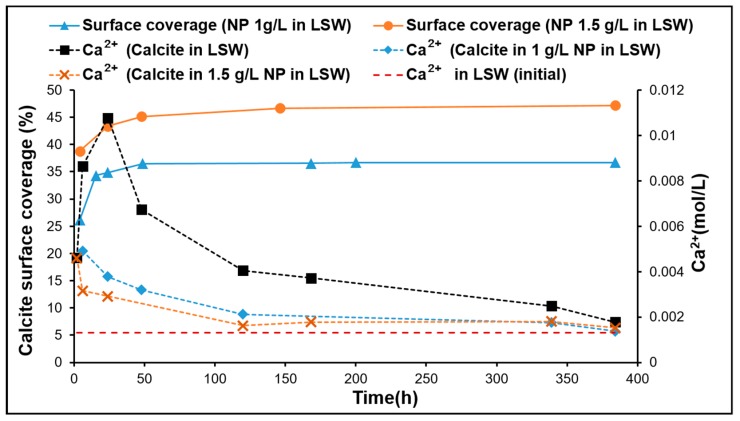
Supernatant Ca^2+^ concentrations with and without nanoparticle and the estimated surface coverage by nanoparticles for LSW fluid.

Figure 6 shows the supernatant Ca^2+^ and surface coverage with nanoparticles as a function of time in the cases of DIW and SSW. The contact barrier between the mineral and DIW is well demonstrated in Figure 6a. When the percentage coverage of the surface by the nanoparticles reached the equilibrium phase, the Ca^2+^ concentrations reached a steady state at about 49 h. The Ca^2+^ concentration was reduced (from ≈0.003 to ≈0.0015 mol/L) by about 50% with nanoparticle adsorption. In the case of SSW, Figure 6b demonstrates a reduction in Ca^2+^ (≈ 0.0046 to 0.0041) of about 10% after 16 h, when the adsorption of the nanoparticle reached equilibrium for the percentage calcite surface coverage of about 27%. It is interesting to observe that the Ca^2+^ concentrations decline rather than increase as a result of the solubility. Figure 7 for LSW (1 and 1.5 g/L nanoparticle concentration) shows similar observations as for SSW. The Ca^2+^ concentrations decline after a concentration spike (without nanoparticles) reaching ≈0.011 mol/L compared to ≈0.0046 mol/L (with nanoparticles). The two most important observations are that Ca^2+^ shows declining trends in both cases, LSW and SSW, as well as a higher initial spike in Ca^2+^ concentration in the case of LSW compared to that of SSW. The reduction trend of Ca^2+^ is difficult to explain. However, there are two possible mechanisms. The first is adsorption of Ca^2+^ onto the silica surface according to the following equation [59]:

(18) $$2\mathrm{SiOH}+{\mathrm{Ca}}^{2+}⇌{\left(-{\mathrm{SiO}}^{-}\right)}_{2}{\mathrm{Ca}}^{2+}+2H^{+}.$$

Equation (18) could support the reduction in Ca^2+^. However, Janusz, Patkowski, and Chibowski [59] previously measured the Ca^2+^ uptake by silica in solutions of ionic strength similar to the LSW used in the present study. They estimated an uptake capacity of ≈0.0016 μmol/L at a pH of 8. This reduction is much lower compared to the reductions in Ca^2+^ concentrations in this study. Therefore, the uptake of calcium is not expected to be the main contributor to the observed Ca^2+^ declining trend. The second hypothesis could be the formation of CaSO_4_ due to possible reaction with SO_4_^2−^ ions present in both fluid cases (LSW and SSW). At the mineral–solution interface, assuming heterogeneous Ca^2+^ distribution, the solubility product of the CaSO_4_ may be exceeded. The smaller peak in the case of SSW (Figure 6b) may be the result of the higher SO_4_^2−^ ion concentration (65% higher than that with LSW). This would kinetically favor faster removal of Ca^2+^ from the fluid in the form of CaSO_4_, when the thermodynamic solubility product (Ksp) is reached. This may be supported by the case of the DIW, where SO_4_^2−^ is absent. We therefore believe that the second mechanism is the cause of this observation.

**Figure 8:**
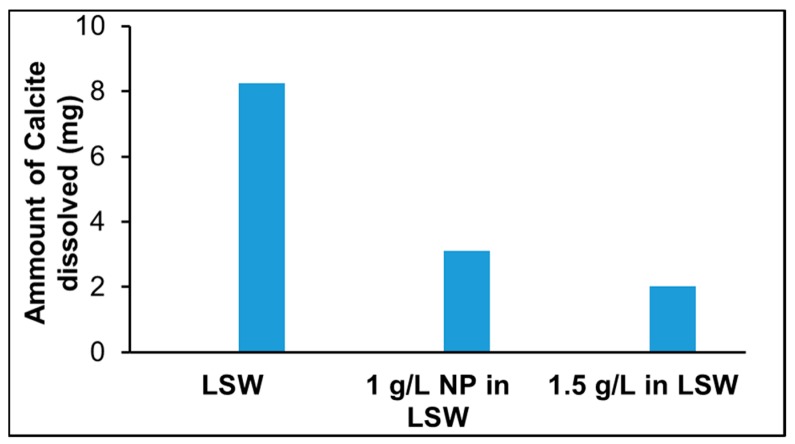
Amount on Calcite dissolved in LSW and with nanoparticle adsorption on calcite.

Figure 7 shows that as the nanoparticle concentration in LSW was increased from 1 to 1.5 g/L, the Ca^2+^ concentration was further reduced at the onset of nanoparticle adsorption. As the adsorption progresses, the Ca^2+^ concentration for the case of 1.5 g/L almost reached the same concentration as in the case of 1 g/L. Near the end of the experiment, in both cases the Ca^2+^ concentration reached a level close to the Ca^2+^ concentration in LSW. The observed decrease in Ca^2+^ concentration may be related to the intraparticle diffusion phenomenon (discussed earlier) occurring after reaching the maximum calcite surface coverage by the nanoparticles. In both cases, Ca^2+^ concentration reduction continues (Figure 7) reaching the lowest Ca^2+^ concentration almost at the same rate until it reached the level of Ca^2+^ concentration in LSW. The Ca^2+^ concentration after the nanoparticle surface coverage reached maximum (about 49 h, Table 4) was about 1.3 times higher for nanoparticle at 1 g/L (≈0.0032 mol/L) than that for 1.5 g/L (≈0.0024 mol/L). The amount of calcite dissolved was estimated from the areas under the produced Ca^2+^ concentration curves in Figure 7 (with and without nanoparticles). The results are shown in Figure 8, where it demonstrates that an increasing nanoparticle concentration led to a lower amount of calcite dissolution. This can have profound implications when designing LSW flooding of chalk reservoirs.

## 4. Conclusions

This study addressed the kinetics of silica nanoparticle adsorption dispersed in three saline waters (DIW, SSW, and LSW). Additionally, the dynamic calcite dissolution related to the progression of nanoparticle adsorption was addressed. On the basis of the observation and analysis made in this study, the following conclusions were made:

1. The adsorption of silica nanoparticles on calcite is best described with a pseudo-second-order model.
2. Both the rate of adsorption and the level of equilibrium adsorption increase substantially as the salinity increases from DIW to SSW.
3. The reduction by half of *R_i_* in LSW as the nanoparticle concentration increases from 1 to 1.5 g/L may be explained by repulsive forces among the nanoparticles as they diffuse from the bulk fluid towards the calcite surface. This may also explain the lower adsorption rate observed for LSW with nanoparticles at 1.5 g/L during the investigation of adsorption kinetic order. The almost equal *R_i_* in both DIW and LSW (1.5 g/L) supports the above hypothesis; where the presence of salt ions (in the LSW) acts as a barrier reducing the adsorption rate, and in the absence of salt ions (in the DIW), the repulsive forces among nanoparticles reduce the adsorption rate.
4. The estimation of the surface forces based on the DLVO theory showed that with nanoparticles in LSW, the interaction between nanoparticles and calcite mineral is less attractive in comparison with SSW and DIW.
5. Adsorption of silica nanoparticles reduces calcite dissolution. This was confirmed by the Ca^2+^ ion concentration, pH, and lower dissolution observed at increased nanoparticle concentrations. Mass balance based on the analyzed Ca^2+^ profile demonstrates the increased dissolution reduction with increasing nanoparticle concentration. This is an important outcome especially when LSW is a candidate for EOR in chalk fields, where less formation damage/dissolution of chalk is expected when silica nanoparticles are combined with LSW.

## Figures and Tables

**Table 1:** Ionic composition of brines.

Ion	Synthetic Seawater (SSW) (mol/L)	Low Salinity Water (LSW) (mol/L)
HCO^3−^	0.002	0.0002
Cl^−^	0.525	0.0525
SO_4_^2−^	0.0240	0.0024
Mg^2+^	0.045	0.0045
Ca^2+^	0.013	0.0013
Na^+^	0.450	0.045
K^+^	0.010	0.0010

**Table 2:** Particle size and Zeta potential of silica nanoparticles and calcite mineral. Deionized water—DIW.

Material	Dispersing Fluid	Temperature (°C)	Hydrodynamic Radius (nm)	pH	Zeta-Potential (mV)
Silica nanoparticles	DIW	25	18.76	6.0	−30.7
Silica nanoparticles	DIW	50	19.29	-	-
Silica nanoparticles	DIW	80	19.7	-	-
Silica nanoparticles	LSW	25	18.96	7.2	−12.1
Silica nanoparticles	LSW	50	19.1	-	-
Silica nanoparticles	LSW	80	19.35	-	-
Silica nanoparticles	SSW	25	28.18	7.3	−6.4
Silica nanoparticles	SSW	50	28.77	-	-
Silica nanoparticles	SSW	80	44.06	-	-
Calcite	DIW	25	-	9.62	−23.4
Calcite	LSW	25	-	8.39	−8.0
Calcite	SSW	25	-	7.56	−3.7

**Table 3:** Summary of the fit parameters from the kinetic order data.

**Pseudo-First-Order Model**				
**Fluid**	**Exp *q_e_* (mg/g)**	***R*^2^**	***k*_1_: (1/h)**	**Estimated *q_e_*: (mg/g)**
DIW (nanoparticle conc 1 g/L)	2.41	0.88	0.055	0.312
SSW (nanoparticle conc 1 g/L)	4.75	0.94	0.2132.5	0.971
LSW (nanoparticle conc 1 g/L)	4.4	0.9025	0.1149	1.09319
LSW (nanoparticle conc 1.5 g/L)	4.75	0.9378	0.0066	0.88923
**Pseudo-Second-Order Model**				
**Fluid**	**Exp *q_e_* (mg/g)**	***R*^2^**	***k*_2_: (g/mg h)**	**Estimated *q_e_*: (mg/g)**
DIW (nanoparticle conc 1 g/L)	2.41	0.99	0.73	2.41955
SSW (nanoparticle conc 1 g/L)	4.75	1	2.5	4.76644
LSW (nanoparticle conc 1 g/L)	4.4	1	0.191	4.44
LSW (nanoparticle conc 1.5 g/L)	4.75	0.99	0.11	5.68

**Table 4:** Summary of initial adsorption of intraparticle diffusion model (IPD) model.

Fluid_Nanoparticle Conc.	*C* (mg/g)	*K* (mg/g h^0.5^)	*R_i_*	*t_eq_*(hrs)_Adsorption Characterization
DIW_1.0 g/L	1.8	0.16	0.25	49 (hrs)_ Strong initial adsorption
LSW_1.0 g/L	2.13	0.51	0.52	49(hrs)_Strong initial adsorption
LSW_1.5 g/L	4.29	0.19	0.24	49(hrs)_Strong initial adsorption
SSW_1.0 g/L	4.56	0.036	0.037	16(hrs)_near complete initial adsorption
